# A CMOS Image Sensor Based Refractometer without Spectrometry

**DOI:** 10.3390/s22031209

**Published:** 2022-02-05

**Authors:** Haechang Yang, Sanghoon Shin, Samir Kumar, Dongmin Seo, Sangwoo Oh, Moonjin Lee, Sungkyu Seo

**Affiliations:** 1 Department of Electronics and Information Engineering, Korea University, Sejong 30019, Korea; didgockd123@korea.ac.kr (H.Y.); ghost10s@korea.ac.kr (S.S.); samiratwork@gmail.com (S.K.); 2 Maritime Safety & Environmental Research Division, Korea Research Institute of Ships & Ocean Engineering, Daejeon 34103, Korea; dseo@kriso.re.kr (D.S.); swoh@kriso.re.kr (S.O.); Moonjin.Lee@kriso.re.kr (M.L.)

**Keywords:** refractive index, refractometer, surface plasmon resonance (SPR), CMOS image sensor, smartphone

## Abstract

The refractive index (RI), an important optical property of a material, is measured by commercial refractometers in the food, agricultural, chemical, and manufacturing industries. Most of these refractometers must be equipped with a prism for light dispersion, which drastically limits the design and size of the refractometer. Recently, there have been several reports on the development of a surface plasmon resonance (SPR)-based RI detector, which is characterized by its high sensitivity and simplicity. However, regardless of the prism, an expensive spectrometer is required to analyze the resonance wavelength or angle of incidence. This paper proposes a method that eliminates the need for the prism and other conventional spectrometer components. For this purpose, total internal reflection SPR technology was used on an Ag thin film, and RI analysis was combined with a lens-free CMOS image sensor or a smartphone camera. A finite-difference time-domain (FDTD) numerical simulation was performed to evaluate the relationship between the output power intensity and Ag film thickness for different RIs at three wavelengths of commercial light-emitting diodes (LEDs). The maximum sensitivity of −824.54 RIU^−1^ was achieved with AG20 at an incident wavelength of 559 nm. Due to its simple design and cost effectiveness, this prism-less, SPR-based refractometer combined with a lens-free CMOS image sensor or a smartphone could be a superior candidate for a point-of-care device that can determine the RIs of various analytes in the field of biological or chemical sensing.

## 1. Introduction

The refractive index (RI) is the rate at which the speed of light traveling through a medium decreases, compared with the speed of light in a vacuum. The refractive index *n* is material specific and is related to the relative permittivity and permeability of the material [1]. Except for ferromagnetic materials, most materials have a unique relative permeability. In nonferromagnetic materials, RI is determined by the relative permittivity of the material [2]. The relative permittivity of a material is used to calculate the associated RI informing a material’s critical optical property [3]. RI sensing has been widely employed in various fields, including biomedical, chemical, environmental, and food processing. It is used, for example, to determine the contamination of liquids and to monitor the sugar or alcohol content in beverages [4,5,6]. Most traditional refractometers, i.e., RI sensors, are based on the structural basis of the Abbé refractometer or the Pulfrich refractometer, both invented in the late 19th century [7]. These refractometers determine the change in RI of a material by measuring the difference in the angle of transmitted or reflected light according to Snell’s law [8]. Therefore, the construction of these refractometers requires a prism and lenses, which makes them bulky [9]. Thus, there is a demand to eliminate the shortcomings of refractometers—such as the need for a prism, material exposure, and the high cost—and to develop a simple and accurate method for measuring the refractive index.

Plasmonic waveguide-based sensors for RI sensing applications have been developed to overcome the above limitations of conventional refractometers [4,10,11]. Plasmons are the quanta of the collective oscillations of the free electrons of a metal compound, which propagate through the near-field surface of the metal [12]. A surface plasmon is an electromagnetic wave excited by external radiation at the interface between metal and dielectric [13,14,15]. Most existing surface plasmon resonance (SPR) sensors use the angle of incidence to determine the intensity of reflected light, which requires the use of a precise spectrometer [16,17,18]. Moreover, the SPR waveguide sensor requires a spectrometer to evaluate the intensity difference between the output and input wavelengths [19,20]. Recently, SPR sensors without spectrometers have been developed to analyze images based on a smartphone camera [21,22].

Recently, more affordable and competitive fiber optic refractometers have been realized through the use of intensity-based measurements that eliminate the need for expensive instruments to measure the optical spectrum [23,24,25]. However, because all of these sensors are based on optical fibers, they are fragile and highly susceptible to damage, which limits their usability in the field and makes them expensive.

**Figure 1:**
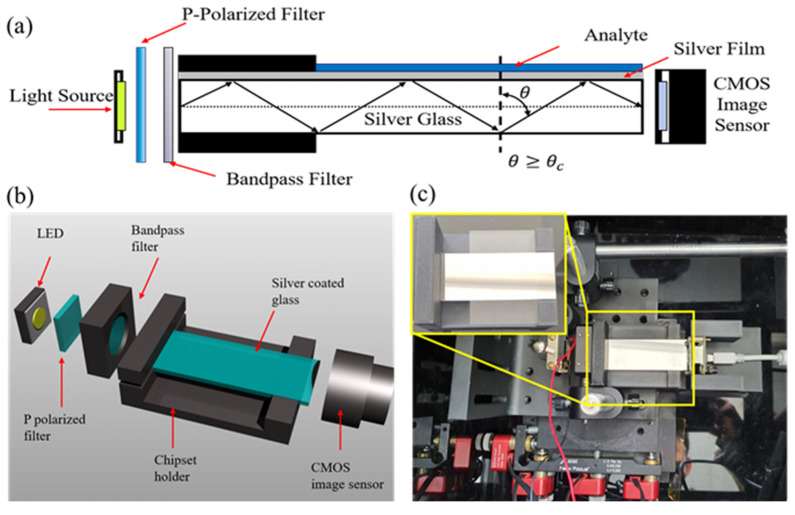
(**a**) Schematic of the surface plasmon resonance refractometer, which consists of simple optoelectronic components—namely, a light-emitting diode (LED), a silver-coated glass substrate, and a CMOS image sensor. The Ag-coated glass acts as a waveguide to generate SPR by total internal reflection; (**b**) a 3D representation of the SPR refractometer; (**c**) photo of the experimental setup for the SPR refractometer.

This article reports the development of a simple, compact, field deployable, and SPR-based RI sensing technique. A simple silver (Ag)-coated glass was employed as a waveguide to generate SPR by total internal reflection. The refractive indices were analyzed by measuring the change in intensity of the output image as a function of the RI of the analyte using a lens-free CMOS image sensor or a smartphone camera. Figure 1 illustrates the proposed SPR refractometer, which consists of simple optoelectronic components—such as a light-emitting diode (LED), a silver-coated glass substrate, and a lens-free CMOS image sensor—enabling the development of a compact, low-cost and field-portable refractometer.

## 2. Materials and Methods

### 2.1. Finite-Difference Time-Domain Simulation

Prior to the experiment, a 2D finite-difference time-domain (FDTD) simulation was performed to figure out the feasibility of implementing an SPR waveguide based on total internal reflection and to find an optimal Ag thickness. FDTD simulation is a widely used technique for solving electromagnetic wave problems and is superior for optical modeling applications. A 2D-FDTD simulation is sufficient for our analysis because the field in the p-polarized incident beam, as well as the plasma oscillations in the metal layer, does not exhibit three-dimensional variation. Additionally, the 2D simulation requires less memory, and the simulation time is shorter. To further simplify the simulation, our simulation geometries were approximations of the real geometries. FDTD simulations were performed using a commercial software package (Fullwave; Synopsys, Mountain View, CA, USA), i.e., a Maxwell equation solver, to investigate the effect of metal layer thickness on SPR generation as described in several SPR-related papers in the literature [26,27,28]. 

**Figure 2:**
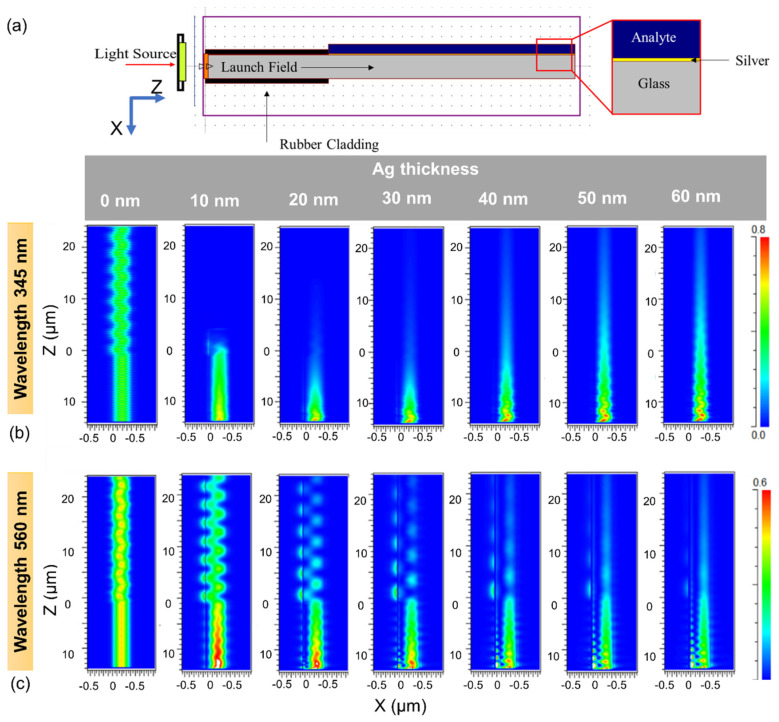
(**a**) Schematic of the surface plasmon resonance RI detector for FDTD simulation. Perfectly matched layers were used in the z-direction to absorb field components at the grid boundaries. Simulation results for different Ag layer thicknesses with (**b**) 345 nm and (**c**) 560 nm wavelength as source. The 0 point on the X and Z axes corresponds to the interface between the Ag film and the glass and the interface between the rubber cladding and the Ag film, respectively. The image above shows the light after it has passed the RI detector. The dark red color indicates the highest intensity value, while the dark blue color indicates the lowest intensity value.

Figure 2 shows the FDTD simulation for different thicknesses of Ag thin film at different incident wavelengths. The size of the Ag thin film sample was 37.5 μm × 0.5 μm, and the Ag thickness varied from 5 nm to 80 nm. The RI of the glass to achieve total internal reflection was assumed to be 1.52. The Ag-coated glass was rubber clad to prevent the incident field from leaking out. The RI of the analyte was varied between 1.33 (the refractive index of water) and 1.47, while the wavelength of the incident field varied between 300 nm and 650 nm. The TM polarized incident light was launched from in the positive z-direction, and perfectly matched layer (PML) boundary conditions were used to block external effects. For this simulation, we performed a parameter sweep over the source incidence angle. The dispersion model of silver is based on the data of the study by Johnson and Christy and can be found in the material library of the software [29,30].

For the simulation, 345 nm and 560 nm light waves were used as excitation sources. The simulation results for different Ag film thicknesses are shown in Figure 2b,c. There is no evidence of SPR absorption in a 0 nm thick Ag film. However, the Ag film exhibits a high level of absorption. 

### 2.2. Fabrication of Ag-Thin-Film-Coated Glass

An Ag-thin-film-coated glass was fabricated according to FDTD simulation results. The Ag thin film was deposited on a glass slide 76 mm × 26 mm × 1 mm by e-beam evaporation with 99.9% Ag pellets at 700 W DC power. The chamber pressure during growth was better than 10^−6^ Torr. Ag thin films with thicknesses of 10, 20, 40, 60, and 80 nm were deposited at a growth rate of 10 nm/s, and hereafter, the samples are referred to as AG10, AG20, AG40, AG60, and AG80, respectively. A small portion of the Ag-coated glass was etched with a chrome etchant, and the thickness of the deposited Ag thin film was verified using an Alpha Step IQ surface profilometer (KLA-Tencor, Milpitas, CA, USA).

### 2.3. Analyte for Refractive Index Measurements

Standard liquid RI solutions (Cargille Labs, Cedar Grove, NJ, USA) with refractive indices of 1.35, 1.37, 1.40, 1.44, and 1.47 were used as reference samples. The RI of water–ethanol and water–acetone solutions changes with the amount of water in the solution [31,32]. A series of water–ethanol and water–acetone sample solutions with different RI values (1.35–1.47) were prepared by changing the mixing ratio of DI water with ethanol or acetone (Samchun Chem., Seoul, Korea). The RI of each sample solution was determined using a refractometer (CAS Co., Seoul, Korea) for each ratio and compared with the reference data.

### 2.4. Experimental Setup

SPR-based refractometers use resonant excitation of surface plasmon waves to detect minute variations in the refractive index near the interface. At the interface between metal and dielectric, surface plasmons are generated at a certain wavelength and angle of incidence and produce an evanescent wave between the interfaces [12]. The wave vector *k_sp_* of the surface plasmon is calculated using the following equation:

(1) $$k_{sp}=\frac{2\pi }{\lambda }\sqrt{\frac{n_{d}^{2}n_{m}^{2}}{n_{d}^{2}+n_{m}^{2}}}$$

where *λ* is the wavelength, *n_d_* is the refractive index of the dielectric medium, and *n_m_* is the refractive index of the metal. Equation (1) can be rewritten as follows:

(2) $$k_{sp}=\frac{2\pi }{\lambda }\sqrt{\frac{\varepsilon_{d}\varepsilon_{m}}{\varepsilon_{d}+\varepsilon_{m}}}$$

where *ε_d_* is the dielectric constant of the dielectric medium, and *ε_m_* is the dielectric constant of the metal. The values *n_m_* and *ε_m_* are complex and is given by the following:

(3) $$\varepsilon_{m}=\varepsilon_{m}^{\prime }+i\varepsilon_{m}^{\prime \prime }$$

(4) $$n_{m}=n_{m\;}^{\prime }+in_{m\;}^{\prime \prime }$$

where $n_{m\;}^{\prime },\;\varepsilon_{m}^{\prime }$ are the real part; $n_{m\;}^{\prime \prime }$, $\;\varepsilon_{m}^{\prime \prime }$ are the complex parts of the refractive index and the dielectric constant, respectively.

The incident light wave that can reach the interface between the metal and glass medium is called the evanescent wave. The wave vector component of this evanescent wave at the interface is

(5) $$k_{z}=\frac{2\pi }{\lambda }n\sin\theta$$

where and *θ* express the incidence angle of the incident light, and *n* represents the dielectric constant of glass.

SPR occurs only when the wavevector of the surface plasmon wave coincides with the wave vector of the evanescent wave generated by the total internal reflection between the metal and the glass medium. At the SPR condition, the electromagnetic fields of evanescent waves and surface plasmons become strongly coupled, resulting in rapid absorption of some of the incident light energy. As a result, the intensity of the reflected light decreases, leading to a significant reduction in transmission intensity. 

From Equation (2), we can deduce that the SPR position also depends on the refractive index of the dielectric medium. When the SPR phenomenon occurs, a part of the incident light is absorbed. Therefore, when the refractive index changes due to a change in the analyte, the SPR position also changes and will decide how strongly it absorbs the incident light. Thus, the intensity distribution of transmitted light on the CMOS camera changes in response to changes in the refractive index of the dielectric medium. We designed our sensor to be angle independent because it measures the output intensity regardless of the SPR position. When the sensor is near the SPR resonance, the output intensity decreases significantly. Since the position of the SPR changes as the RI of the medium changes, the change in output intensity can be quantified as a function of the RI change. 

In this study, total internal reflection in the form of a waveguide was used to reduce the dependence on the angle of incidence and to observe the changes in the refractive indices of the dielectric material on the metal surface. 

Figure 1b shows a schematic diagram of the refractometer setup. A glass slide (76 mm × 26 mm × 1 mm) was used to realize the total internal reflection. Black covers were used on the top and underneath the glass to block all-penetrating light except for the side illumination. An M365 L2 LED (Thorlabs, Newton, NJ, USA) with peak emission at 365 nm and 1.5 mW optical power and broadband LED SAWS1566A (SunLike LED; Seoul Semiconductor, Seoul, Korea) were used in conjunction with MF475 and MF559 bandpass filters (Thorlabs, Newton, NJ, USA) to emit light at 475 nm and 559 nm with an optical power of 3.93 μW and 5.37 μW, respectively. The critical angle between the inside of the substrate and the glass surface was calculated to be 42° using Snell’s law. The light source was incident almost parallel to the Ag layer, with an incident angle of about 90°. This geometry confirmed that all light sources illuminated from the side had a total internal reflection, as it was larger than the critical angle.

A polarizing filter (Edmund Optics, Barrington, NJ, USA) was placed between LED and the bandpass filter to produce P-polarized light for SPR. The chipset and image sensor mounts were printed using the Rhino 5 3D design tool and a 3D printer. An 8-bit pixel depth CMOS monochrome USB camera (EO-0512M; Edmund Optics, Barrington, NJ, USA) (with gain 50 and offset 0) or a Samsung Galaxy Note 10+ smartphone camera (with ISO 200, shutter speed 1/1000, and aperture F1.5) was used as the detector. CMOS image sensors have a compact size and low power consumption because they do not require a separate circuit such as the charge-coupled device (CCD) image sensor. CMOS image sensors have recently been used as camera sensors in mobile products such as smartphones and digital cameras. The image sensor and chipset were aligned using a three-axis microblock stage. Prior to the experiment, a black matrix, i.e., shrink tubing, was used to encase the silver-coated glass. A liquid analyte was uniformly placed by placing the coverslip on top of the silver-coated glass and analyte.

## 3. Results and Discussion

### 3.1. FDTD Simulation Results

**Figure 3:**
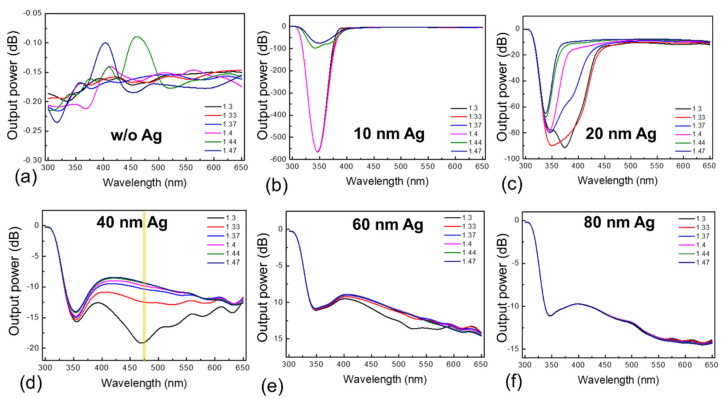
The results of FDTD simulation of output power at wavelengths in the range of 300–650 nm for (**a**) glass without thin film, (**b**) AG10, (**c**) AG20, (**d**) AG40, (**e**) AG60, and (**f**) AG 80 samples with Ag thickness of 0, 10, 20, 40, 60, and 80 nm.

Figure 3 shows the simulation results for various Ag thin film thicknesses at wavelengths in the range of 300 nm to 650 nm that were studied. No SPR excitation or resonance-induced power absorption was observed without the Ag thin film (W/O Ag, Figure 3a). As a result, the variation of the output field caused by different RIs was not significant. For sample AG10, RI values below 1.4 resulted in a drastic drop in output power at approximately 350 nm, which can be attributed to SPR. Meanwhile, RI values above 1.4 showed a broad and less intense absorption.

In addition, no change in absorbance was observed at wavelengths above 400 nm for any RI value. Samples AG20, AG40, and AG60 showed strong absorption at approximately 350 nm for all RI values and variable absorption at wavelengths above 400 nm, depending on the observed RI values and Ag film thickness. The absorption peaks of sample AG20 shifted as a function of RI values; however, there was no significant change in absorption at wavelengths longer than 450 nm. Sample AG80 showed shallow absorption over the entire wavelength range of 350–650 nm, and the absorption intensity was not affected by the RI value. This is because, as the thickness of the Ag layer increases, the evanescent field continues to decrease, resulting in nonzero reflectance [33]. Analysis of the output power as a function of wavelength, and Ag thin film thickness revealed that sample AG40 is best suited for RI measurement because it exhibits selective absorption over the entire wavelength range of 350–650 nm and the absorption spectrum shift as a function of the RI of the solution under test. Furthermore, since sample AG40 appeared to have a well-defined wavelength window (at the desired wavelength of 475 nm) for various RI values, this sample, with the measurement range shown in Figure 3d, was selected for better resolution.

**Figure 4:**
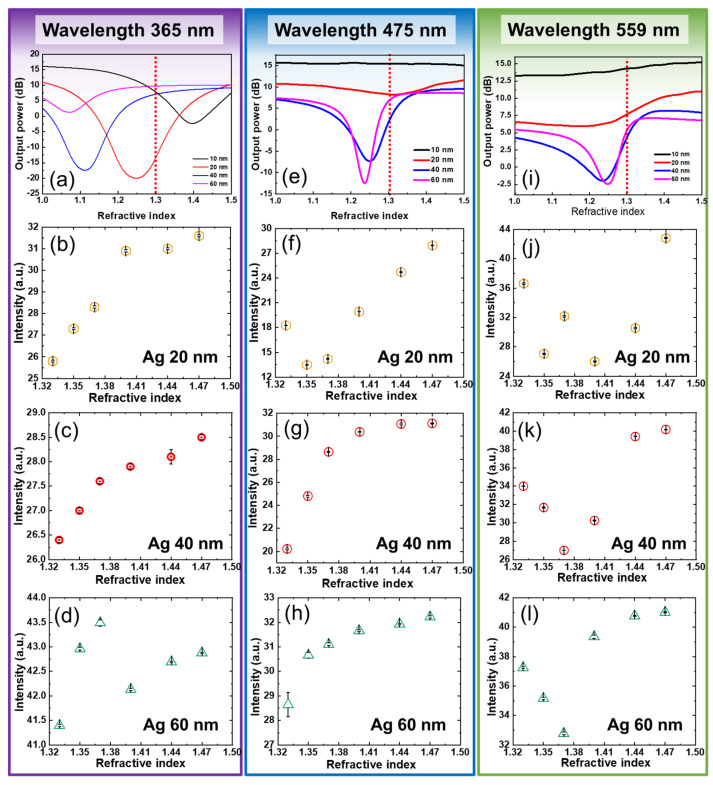
(**a**,**e**,**i**) show the numerical output power variation as a function of the RIs for various Ag thickness at the illumination wavelengths of 365 nm, 475 nm, and 559 nm, respectively. Figure sets (**b**–**d**), (**f**–**h**), and (**j**–**l**) illustrate the experimental output intensity variation as a function of RIs for the samples of AG20, AG40, and AG60 at the illumination wavelengths of 365 nm, 475 nm, and 559 nm, respectively. Sample AG40 alone exhibited a monotonic increase in initial intensity at wavelengths 365 nm and 475 nm.

Based on the above FDTD simulation results, the wavelengths of 365, 475, and 559 nm, i.e., the dominant wavelengths of commercially available LEDs, were selected to determine the refractive indices of the Ag films with thicknesses of 20, 40, and 60 nm. Figure 4a,e,i show the variation of output power as a function of the refractive index for each wavelength. At a given wavelength, significant absorption dips were observed for films of different thicknesses. For RI values above 1.33, there was no significant difference in intensity for 475 nm and 559 nm light sources for sample AG20. There were also no significant differences in output power between samples AG40 and AG60 at 365 nm; however, there was a significant decrease in output power at 475 nm and 559 nm.

Thin Ag films show significant differences in optical and dielectric properties, compared with bulk material [34]. Performing FDTD calculations with an exact thickness equal to the observed thickness would not provide physical insight into the role of Ag thin film thickness. Comparing the results of such an investigation with those of an experiment would provide a test of validity, but that was not the goal of our simulation. The simulations described in this article were performed to investigate the effects of Ag film thickness on absorption by SPR and to determine the optimal Ag film thickness for initial RI detection. The main result is that the absorption is significantly enhanced in thin Ag films with a thickness of 20–60 nm, indicating that these samples can be used for further experimental studies.

### 3.2. Experimental Results of the Refractive Index of the Liquid

Samples AG20, AG40, and AG60 were used for RI measurements with incident light at 365, 475, and 559 nm. FDTD simulation indicated that samples AG10 and AG80 had poor RI sensing performance. Therefore, they were removed from the study. The volume of each analyte was kept constant at 200 μL. Figures S1–S3 of the electronic supplementary information (ESI) show the images of analytes with different RI values taken with a CMOS camera at 365, 475, and 559 nm, respectively. ImageJ, an open image processing software provided by the NIH, was used to analyze the intensity of these images. Figure 4b–d show the variation in intensity as a function of RIs for samples AG20, AG40, and AG60 at 365 nm, respectively. The output intensity increased with increasing RI for samples AG20 and AG40, consistent with the simulation results. However, as RI increased, no increase in output intensity was observed for sample AG60. Figure 4f–h show the variation in intensity as a function of RIs for samples AG20, AG40, and AG60 at 475 nm, respectively. For samples AG40 and AG60, each output intensity increases with an increase in RI values. However, for AG20, the intensity first decreases, and then, after the RI value of 1.37, increases. The trend in output intensities for samples AG40 and AG60 is reasonably consistent with the simulation results. Figure 4j–l illustrate the change in output intensity for AG20, AG40, and AG60 at 559 nm, respectively. Compared with 365 nm and 475 nm, no apparent relation between the output power and RI was observed at 559 nm. Thus, the output intensities at various RIs, e.g., RI = (1.33–1.47), were relatively strong on the 40 nm thick Ag-coated glass under 475 nm illumination. Figure 4g shows the figure of merit; this condition also exhibited a monotonic increase in the output intensity at the given RI range. This result is qualitatively consistent with the FDTD simulation results in Figure 3d.

The sensitivity (*S*) of an SPR sensor is defined as the ratio between the change in the resonance angle (∆*θ*_res_) and the change in refractive index (Δ*n*), i.e., *S* = (∆*θ*_res_/Δ*n*) in refractive index units (RIUs). Similarly, we defined the sensitivity of our system as the change in output intensity per unit change in the refractive index of the sensing medium. The maximum sensitivity of −824.54 RIU-1 was obtained using AG20 at an incident wavelength of 559 nm. Sensitivity data at all RI values for AG20, AG40, and AG60 for the three wavelengths can be found in Table S1 (ESI).

### 3.3. Measurements of the Refractive Index of Ethanol–Water and Acetone–Water Solutions

**Figure 5:**
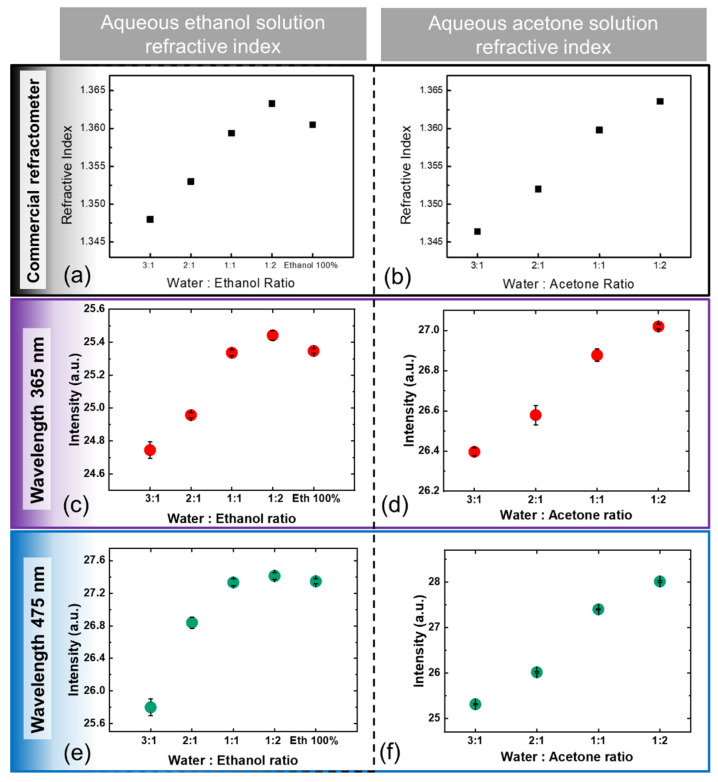
The refractive index of (**a**) a water–ethanol solution and (**b**) a water–acetone solution was measured using a commercial refractometer. The output intensity of CMOS image sensor-based RI detection for different mixing ratios of ethanol and water with (**c**) 365 nm and (**e**) 475 nm and different mixing ratios of acetone and water with (**d**) 365 nm and (**f**) 475 nm.

Based on the results of the refractometric studies described above, RI detection of ethanol–water and acetone–water solutions was performed using sample AG40 at wavelengths of 365 nm and 475 nm (Figure 5). Although the AG40 sample did not have the best sensitivity, it showed a linear relationship with changes in RI, which is another important property and can be used to define the calibration function. The RI was measured using the proposed refractometer for different combinations of ethanol/acetone–water mixing ratios (Figure 5c–f) and compared with a commercial refractometer (Figure 5a,b). Figure 5 shows that the trends of the output intensity of the proposed device for different RI values from different chemical mixtures agree well with the measurement results of the reference refractometer. 

### 3.4. Smartphone Camera-Based Refractive Index Detection

**Figure 6:**
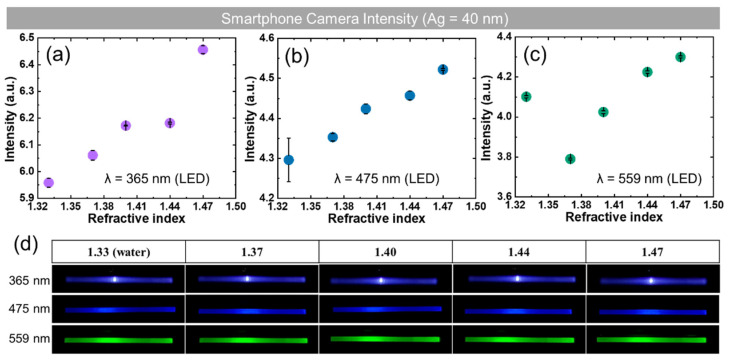
The result of smartphone camera-based RI detection system: (**a**) experimental result data; (**b**) the resulting image of smartphone camera-based RI detection system. (**a**–**c**) show the output intensity as a function of RIs for wavelengths of 365, 475, and 559 nm obtained from (**d**) images captured with a smartphone camera.

Sample AG40 was used for RI detection with a smartphone camera (Galaxy Note 10+; Samsung, Seoul, Korea). Figure 6a–c show the output intensities as a function of RIs between 1.33 and 1.47 for illumination wavelengths of 365, 475, and 559 nm. Figure 6d shows the raw images acquired under each condition. As can be seen in Figure 6, the best results were observed with linearity between output intensity and RI with 475 nm for the RI region. When illuminated at a wavelength of 559 nm, a dip was observed at an RI of 1.37 with the smartphone camera (Figure 6c) and was identical to the result obtained using the lens-free CMOS image sensor (Figure 4k). The result at a wavelength of 365 nm was comparable to that of the CMOS image sensor experiment, although a comparatively low value was obtained at a refractive index of 1.44. This observation that the response of the sensor depends on the wavelength is consistent with previous reports [35]. Therefore, the proposed device and technique can easily measure the RI changes with a lens-free CMOS image sensor or smartphone camera.

## 4. Conclusions

In summary, we presented a simple, low-cost, and portable SPR-based refractometer capable of measuring refractive indices using a 10–80 nm thick Ag thin film of coated glass and a lens-free CMOS image sensor or smartphone camera. Unlike conventional refractometers, the proposed platform does not require a bulky prism or other spectroscopy components that would severely limit its applications. FDTD simulations confirmed the feasibility of implementing an SPR waveguide with total internal reflection and were used to determine the appropriate Ag thickness and LED illumination wavelengths for RI detection in the range 1.33–1.47. The numerical and experimental results show that a 40 nm thick Ag layer at an illumination wavelength of 475 nm provides the best RI detection performance with a strong and stable output signal. In the RI detection experiments of different chemical mixtures, the output intensity trends of the proposed device agreed well with the measurement results of the reference refractometer, regardless of the type of image sensor. Due to its simple design and cost effectiveness, the proposed SPR-based refractometer combined with a lens-free CMOS image sensor, or a smartphone can be an excellent candidate for a point-of-care device that enables RI detection of various analytes in the fields of biological or chemical sensing.

## Data Availability

The underlying data of the results of this paper are not publicly available at this time but may be obtained from the authors upon reasonable request.

## Supplementary Materials

The following supporting information can be downloaded at: https://www.mdpi.com/article/10.3390/s22031209/s1, Figure S1: Captured images for different RIs by a monochromatic lens-free CMOS image sensor with different Ag film thickness under 365 nm LED illumination. (a) Ag film of 20 nm, (b) Ag film of 40 nm, and (c) Ag film of 60 nm, Figure S2: Captured images for different RIs by a monochromatic lens-free CMOS image sensor with different Ag film thickness under 475 nm LED illumination. (a) Ag film of 20 nm, (b) Ag film of 40 nm, and (c) Ag film of 60 nm, Figure S3: Captured images for different RIs by a monochromatic lens-free CMOS image sensor with varied Ag film thickness under 559 nm LED illumination. (a) Ag film of 20 nm, (b) Ag film of 40 nm, and (c) Ag film of 60 nm, Table S1: Sensitivity for different thicknesses of Ag films at different wavelengths, Table S2: RI values calculated from intensity measurements of aqueous ethanol/acetone solutions at different concentrations.
