# Supplementary material for: A CMOS Image Sensor Based Refractometer without Spectrometry
Source: Sensors (Basel). 2022 Feb 5;22(3):1209. doi: 10.3390/s22031209 (PMC8840477; doi:10.3390/s22031209)
Supplement: Supplementary file 1 [file sensors-22-01209-s001.zip › sensors-1536687-supplementary.pdf]

Supplemental Information

(a) Wavelength 365nm, Ag 20nm

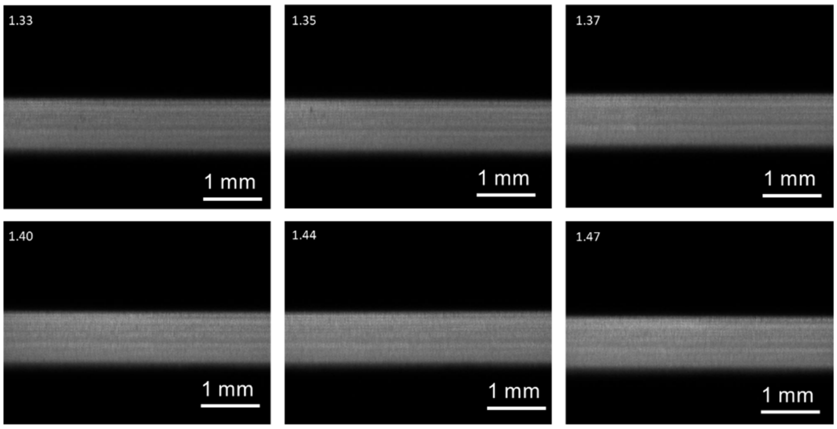

(b) Wavelength 365nm, Ag 40nm

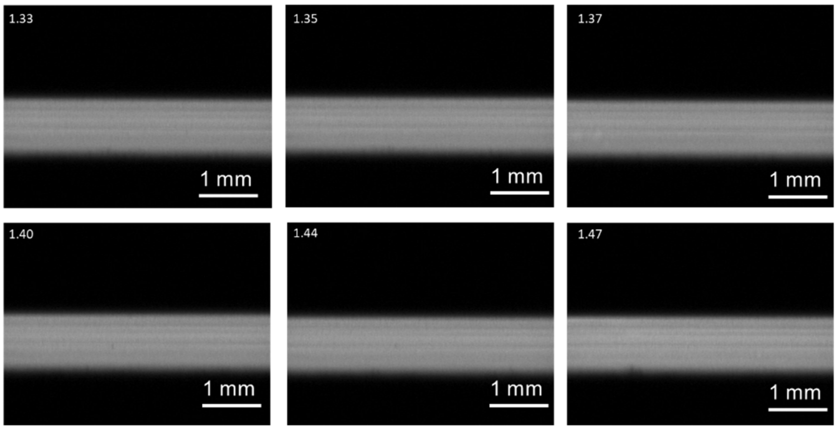

(c) Wavelength 365nm, Ag 60nm

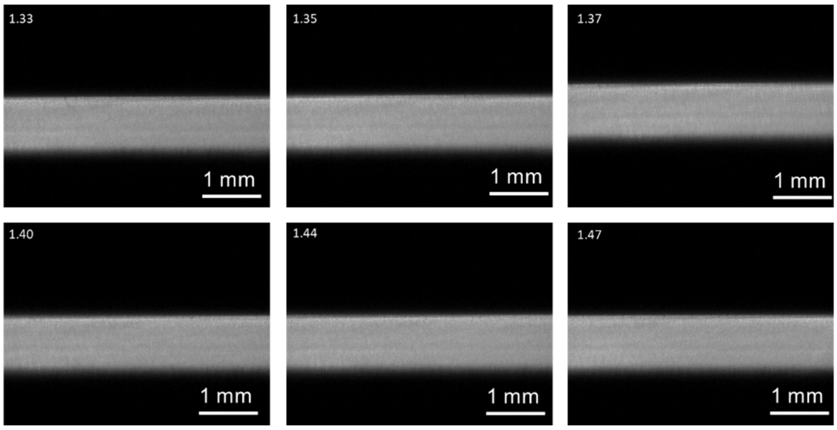

Figure S1. Captured images for different RIs by a monochromatic lens-free CMOS image sensor with different Ag film thickness under 365 nm LED illumination. (a) Ag film of 20 nm, (b) Ag film of 40 nm, and (c) Ag film of 60 nm.

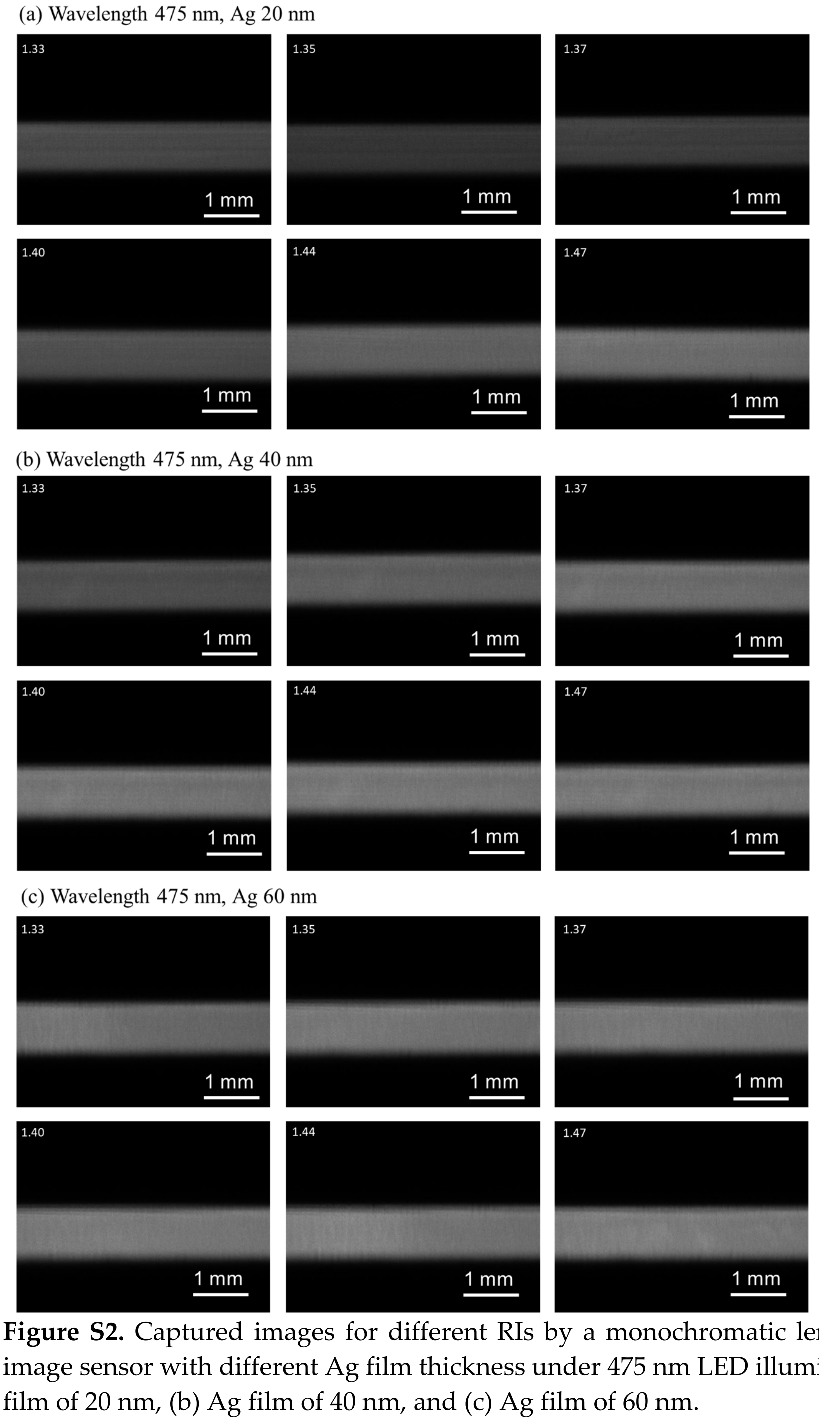

Figure S2. Captured images for different RIs by a monochromatic lens-free CMOS image sensor with different Ag film thickness under 475 nm LED illumination. (a) Ag film of 20 nm, (b) Ag film of 40 nm, and (c) Ag film of 60 nm.

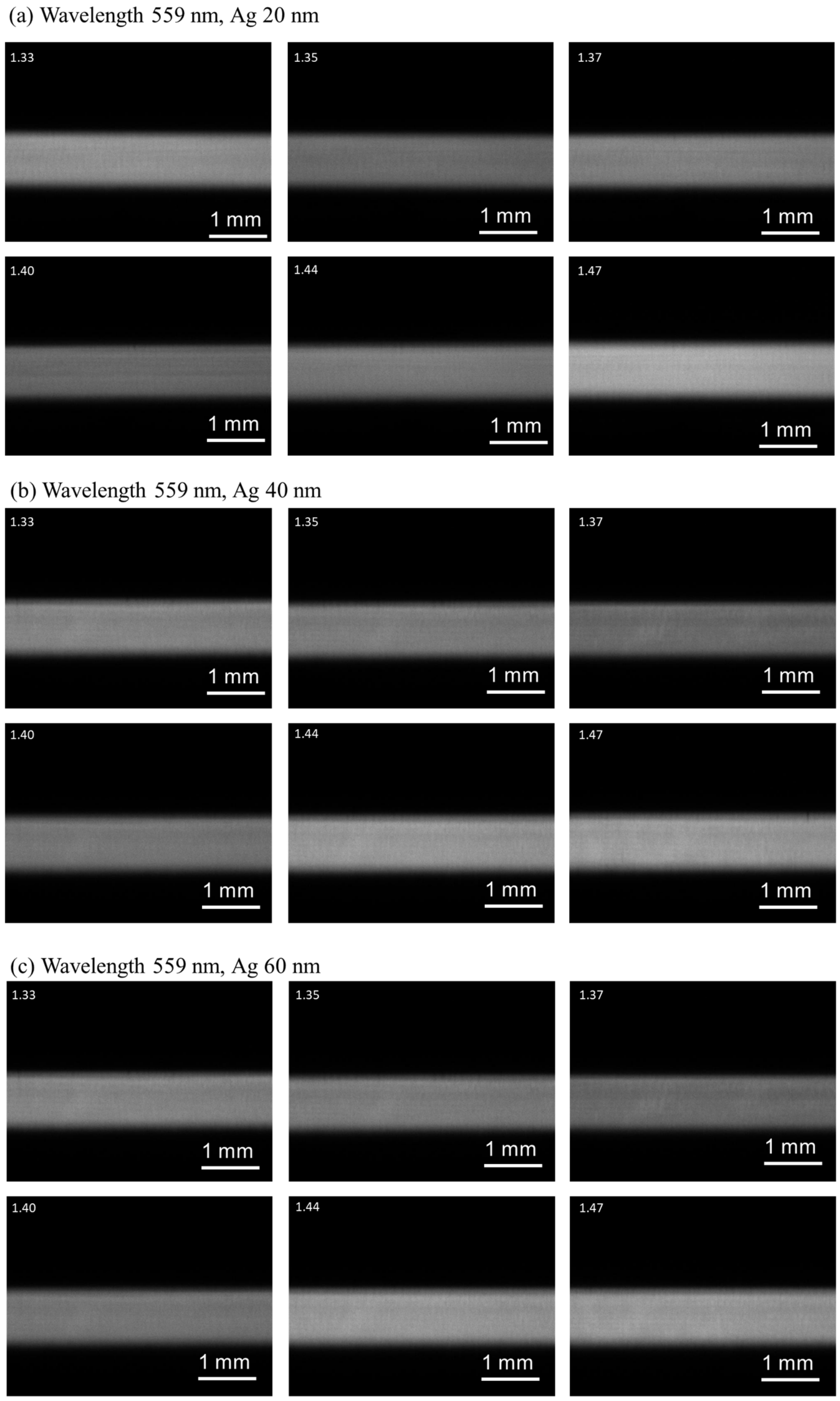

Figure S3. Captured images for different RIs by a monochromatic lens-free CMOS image sensor with varied Ag film thickness under 559 nm LED illumination. (a) Ag film of 20 nm, (b) Ag film of 40 nm, and (c) Ag film of 60 nm.

Table S1. RI values calculated from intensity measurements of aqueous ethanol/acetone solutions at different concentrations.

Refractive index	Thickness of Ag layer								
	**20 nm**			**40 nm**			**60 nm**		
	Wavelength (nm)			Wavelength (nm)			Wavelength (nm)		
	365	475	559	365	475	559	365	475	559
	Sensitivity			Sensitivity			Sensitivity		
1.33	**90.25** (0.05)	**238.67** (0.32)	***-824.54*** (1.09)	**33.18** (0.04)	**198.99** (0.21)	-64.46 (0.29)	**101.81** (0.09)	**134.11** (0.18)	-98.72 (0.04)
1.35	77.31 (0.05)	36.08 (0.04)	-112.04 (1.76)	32.31 (0.02)	168.49 (0.28)	-166.75 (0.36)	52.4. (.015)	62.56 (0.26)	**-112.20** (0.05)
1.37	65.26 (0.03)	191.02 (0.03)	30.51 (1.23)	8.1 (0.03)	133.18 (0.28)	-40.82 (0.34)	-2.16 (.019)	17.32 (0.03)	12.53 (1.25)
1.40	41.61 (0.05)	145.38 (0.03)	-72.72 (1.07)	6.53 (.03)	77.24 (0.31)	**177.38** (0.26)	-19.61 (0.18)	12.86 (0.03)	46.75 (0.08)
1.44	14.80 (0.06)	119.40 (0.28)	307.22 (0.91)	12.92 (.04)	22.08 (0.31)	121.53 (0.24)	9.35 (0.27)	8.04 (0.03)	19.95 (0.09)
1.47	6.25 (0.06)	107.7 (0.03)	530.43 (0.71)	-2.14 (.04)	-44.11 (0.32)	33.23 (0.30)	3.66 (0.23)	4.63 (0.03)	-0.34 (0.07)

Table S2: RI values calculated from intensity measurements of aqueous ethanol/acetone solutions at different concentrations.

Ratio	Aqueous ethanol solution			Aqueous acetone solution		
	**Commercial refarctometer**	**365 nm**	**475 nm**	**Commercial refarctometer**	**365 nm**	**475 nm**
3:1	1.348	1.327	1.280	1.347	1.332	1.233
2:1	1.353	1.339	1.332	1.352	1.341	1.267
1:1	1.359	1.359	1.356	1.360	1.356	1.335
1:2	1.363	1.365	1.360	1.364	1.363	1.364
100 % (ethanol/acetone)	1.360	1.359	1.357			
